# Emulsion templating of PCL:PGS methacrylate blends for soft tissue engineering

**DOI:** 10.3389/fbioe.2026.1758159

**Published:** 2026-03-19

**Authors:** Caitlin S. Ryan, Christopher E. F. Barkshire, Maria F. Velázquez de la Paz, Gwendolen C. Reilly, Frederik Claeyssens

**Affiliations:** 1 School of Chemical, Materials and Biological Engineering, University of Sheffield, Sheffield, United Kingdom; 2 Insigneo Institute for In-Silico Medicine, University of Sheffield, Sheffield, United Kingdom

**Keywords:** emulsion templating, polymer blend, porous, soft tissue engineering, tissue engineering

## Abstract

**Introduction:**

Polycaprolactone (PCL) and poly (glycerol sebacate) (PGS) are biodegradable polymers with which high internal phase emulsion (HIPE) templating may be used to create highly porous structures. Although both polymers have been reported for a wide range of hard- and soft-tissue applications, several challenges remain. For example, PCL structures require surface treatment to allow for efficient cell infiltration, which becomes difficult with thick, complex geometries, and PGS is a soft polymer which results in the collapse of its porous structure during necessary processing steps.

**Methods:**

Here, we demonstrate how methacrylated forms of PCL (PCL-M) and PGS (PGS-M) can be blended to create highly tailorable porous structures that support cell growth and overcome the limitations of the two polymers individually. Material properties were characterised via mechanical testing, porous structures were imaged via scanning electron microscopy, and biological properties were investigated by resazurin assay and fluorescent staining.

**Results and Discussion:**

Mechanical testing of bulk blends of PCL-M and PGS-M demonstrated that the polymers mix together uniformly to provide predictable properties at different weight:weight ratios. PolyHIPEs formed of PCL-M and PGS-M were stable and exhibited highly interconnected porosity. Further investigation was undertaken on the 50:50 blend due to its favourable viscosity and rounded porous structure. Adjusting the synthesis parameters of the 50:50 PCL-M:PGS-M blend demonstrated that a wide range of porous structures can be fabricated, with average pore size ranging from 10–69 μm. The blend was demonstrated to be cell compatible, and collagen staining demonstrated that extracellular matrix adhered to the material. Blend-based scaffolds did not require surface treatment to maintain long term cell adhesion, unlike PCL-M-only controls. Overall, this study demonstrated that highly tuneable porous structures for a wide variety of tissue engineering applications can be created by blending PCL-M and PGS-M to form a composite that exhibits tailorable properties in between those currently known for PCL-M and PGS-M alone.

## 1 Introduction

Porous polymeric biomaterials with a high degree of openness and interconnectivity have many benefits in tissue engineering applications. For example, on a cellular scale, connected porosity permits cell migration, ingrowth, and nutrient transport (Furmidge et al., 2023). On a larger scale, porosity can be beneficial for drug encapsulation, tissue ingrowth, and vascularisation (Maksoud et al., 2022).

Several methods are used to create porosity within polymers including salt/porogen leaching, gas foaming, freeze drying, electrospinning, and emulsion templating (Maksoud et al., 2022; Abbasi et al., 2020; Hollister, 2005; Montanheiro et al., 2022). Among these methods, emulsion templating to form polymer high internal phase emulsions (polyHIPEs) is particularly advantageous because of its low cost and ability to create highly tunable porosity using water as a porogen, making it suitable for a wide range of tissue-engineered applications (Jackson et al., 2023). Emulsion templating is the process of mixing two immiscible phases, typically water (the internal phase) and a polymer (the external continuous phase), with an emulsifier to stabilise the emulsion by reducing the interfacial tension between internal phase droplets (Foudazi, 2021). An emulsion is considered to be a polyHIPE once the internal phase is greater than 74.05% of the total volume (Mert and Mert, 2022). The external phase is then solidified through polymerisation, and the dispersed droplets of the internal phase act as a template for the formation of pores. The internal phase is subsequently removed through washing or dissolution techniques, and a highly porous and interconnected structure remains. The average pore diameter in the resulting polyHIPE ranges from microns to tens of microns (Cameron, 2005). Removal of the internal phase is an easy process due to the excellent interconnectivity which is formed as the external phase ruptures at the thinnest sections between droplets of the internal phase (Silverstein, 2014). This reduces the risk of residual particles negatively affecting the structure’s biocompatibility, unlike salt/porogen leaching methods where this is a commonly reported issue (Montanheiro et al., 2022; Tan et al., 2011). PolyHIPEs also have highly tuneable mechanical properties which are easily altered by changing the porous structure (Huš and Krajnc, 2014; Kramer et al., 2023) making them suitable for both hard and soft tissue applications unlike commonly researched electrospun scaffolds which cannot replicate thicker 3D geometries and have notoriously weak compressive strength (Zulkifli et al., 2023).

Of particular interest are polyHIPEs that are made with biodegradable polymers as these can be used for tissue-engineered applications, where an implant does not need to be removed once in place. Two such polymers are polycaprolactone-methacrylate (PCL-M) and poly (glycerol sebacate)-methacrylate (PGS-M). PCL-M is the photocurable form of polycaprolactone (PCL) which is a synthetic polymer that has been approved by the Food and Drug Administration of the United States of America for several uses including drug delivery devices, sutures, and adhesion barriers (Li and LaBarbera, 2017). Porous structures made from PCL have been extensively studied within tissue engineering using a range of approaches. For example, composite natural-synthetic structures made from PCL reinforced with glycosaminoglycans and hyaluronic acid exhibited 68% porosity and supported chondrocyte phenotype cells, demonstrating a promising strategy for fibrocartilage tissue engineering (Borzacchiello et al., 2011). Additionally, patient-specific multiphasic scaffolds that replicate the intervertebral disc structure have been created from PCL via additive manufacturing techniques (Gloria et al., 2020). As a polyHIPE, PCL-M is versatile and can be used for both hard and soft tissue applications with such scaffolds being developed for use as bone implants, wound healing models, nerve guidance conduits and breast cancer models (Jackson et al., 2023; Dikici et al., 2019; Field et al., 2021).

PGS-M is the methacrylated form of poly (glycerol sebacate) which is a comparably softer synthetic polymer than PCL-M. PGS is a hydroxyl-rich polyester whose mechanical properties can be tailored by altering the prepolymer weight, functionalisation density, and crosslinking strategy. This makes it advantageous over other commonly used soft polymers, as tailoring can be achieved without having to modify the backbone chemistry. Additionally, the high density of hydroxyl groups along the polymer backbone enables a wide range of chemical modification strategies. For example, PGS has been functionalised via urethane chemistry (Ma et al., 2021), and methacrylation (Pashneh-Tala et al., 2023), to form tunable elastomers that are mechanically robust. PGS has also been PEGylated to modulate hydrophilicity and biological interactions, further demonstrating its adaptability (Patel et al., 2013; Aleemardani et al., 2022; Aleemardani et al., 2023). Due to the viscoelastic behaviour exhibited by PGS, polyHIPEs formed from PGS-M are thought to be better tailored to soft tissue applications such as vascular grafts (Pashneh-Tala et al., 2023; Vogt et al., 2021).

Previously, the selection of the porous structures of PCL-M and PGS-M polyHIPEs to suit tissue engineering-based applications has been reported. Pore shape, average pore diameter, and interconnectivity can readily be tuned by altering the emulsion’s composition, such as with the addition of solvents to reduce external phase viscosity, and synthesis parameters. These parameters can include internal phase temperature, mixing speed during the addition of the internal phase, and time between emulsion formation and solidification. For example, Furmidge et al. were able to demonstrate that the use of gelatin as an internal phase, rather than the conventional water, created polyHIPE structures with larger pore sizes in both PCL-M and PGS-M (Furmidge et al., 2023). Alternatively, Velazquez et al. demonstrated that altering the solvent type used in PGS-M polyHIPEs affected the interconnectivity and shape of the pores (Velázquez de la Paz et al., 2023).

Even with ideal porous structures, PCL-M and PGS-M polyHIPEs still have disadvantages that limit their use for several tissue engineering applications. For example, PCL-M is hydrophobic which means this material typically requires surface modification to enable rapid cell attachment. This hydrophobicity also reduces the wettability of the porous structures made from PCL-M and, during *in vitro* work, limits cell infiltration (Dikici et al., 2019). Complete surface modification of porous structures is depth-dependent. It has been demonstrated that plasma treatment can homogeneously coat an 85% porous, 3 mm thick disc however increasing the depth beyond this results in uneven coating with the least deposition occurring at the core (Barry et al., 2005; Viswanathan et al., 2014). Conversely, PGS-M is more hydrophilic than PCL-M due to the hydroxyl groups along its backbone allowing for structures that support cell infiltration (Rai et al., 2012; Wang et al., 2002). However, PGS-M is limited by its weak mechanical properties which are not favourable for hard tissue applications (Pashneh-Tala et al., 2023) and its high viscosity in emulsion form, that limits available additive manufacturing techniques such as stereolithography (Ni et al., 2018). The surface treatment of PGS-M is also limited by the collapse of the constructs when being dried at room temperature or freeze-dried (Pashneh-Tala et al., 2023).

One way of improving polymer performance for tissue engineering is by the inclusion of other polymers with complementary properties. For electrospinning, bulk forms of both PCL and PGS have been either blended or coated with other polymers to create scaffolds with more favourable biological properties or tailored mechanical properties (He et al., 2005; Ghasemi-Mobarakeh et al., 2008). For example, PCL has been blended with gelatin to create electrospun scaffolds with improved polymer-cell interactions, although this caused a significant reduction in mechanical properties because of the weak mechanical properties of gelatin (Ghasemi-Mobarakeh et al., 2008). PCL has also been blended with a non-acrylate version of PGS to create a polymer blend with ideal viscosity for electrospinning (Sant et al., 2011). When tested for biocompatibility, the PCL:PGS blended scaffolds showed better cell attachment and proliferation than PCL-only scaffolds (Sant et al., 2011). Despite these options, many established strategies to improve PCL bioactivity rely on surface modification or post-fabrication coatings, which are often limited by poor long-term stability, non-uniform coverage within 3D porous structures, and added processing complexity (Liang et al., 2024; Goreninskii et al., 2022). In contrast, internal polymer blending provides a route to uniformly improve hydrophilicity and biological performance throughout the scaffold structure, making it particularly useful for highly interconnected porous architectures such as polyHIPEs.

Interestingly, the limitations of PCL-M and PGS-M when used to create porous structures appear to oppose the other material’s advantages. Hence it was hypothesised that by blending the two polymers, PCL-M and PGS-M could counteract each other’s limiting properties to create a highly versatile, porous material. For example, PCL-M would be able to provide rigidity to the blend meaning structures would be able to maintain their porous structures without collapsing whilst being dried, unlocking potential applications that require dehydrated storage. PGS-M could provide a hydrophilicity that is needed for cellular interactions, meaning that resulting structures do not need to undergo surface treatments before use.

Here we investigate for the first time how PCL-M and PGS-M can be blended to create tailorable polyHIPE structures with favourable properties for both hard and soft tissue applications. Blends of different ratios were compared to show the tunability of the mechanical properties and assess the mixability of the two polymers. The porous structure of the 50:50 blend was then tailored for tissue engineering applications by altering synthesis parameters and characterising the porous structure with scanning electron microscopy (SEM). Finally, the biological properties of the 50:50 blend were tested in comparison to PCL-M-only and PGS-M-only polyHIPEs using both fibroblasts and osteoblasts to represent soft and hard tissue applications respectively.

## 2 Materials and methods

### 2.1 Materials

Pentaerythritol (99%), e-caprolactone (97%), tin (II) 2-ethyl hexanoate, photoinitiator (2,4,6-Trimethylbenzoyl Phosphine Oxide/2-Hydroxy-2-Methylpropiophenone blend), glycerol (99%), sebacic acid (99%), 4-methoxyphenol (99%, MeHQ), triethylamine (99.5%, TEA), methacrylic anhydride (94%, MEA), resazurin sodium salt, Triton X-100, L-glutamine, trypsin-EDTA, penicillin/streptomycin, foetal bovine serum (FBS), Sudan Black, DAPI, 37% formaldehyde (FA) solution, DPX mounting media, and haematoxylin solution were purchased from Sigma Aldrich (Poole, United Kingdom). Dichloromethane (99%, DCM), hydrochloric acid (37%, HCl), chloroform (99%), toluene (99.5%), ethanol (99%), methanol (99%), phosphate buffered saline (10X solution, PBS), Quant-iT dsDNA high sensitivity assay kit and Dulbecco’s modified eagle medium (DMEM) were purchased from Fisher Scientific (Pittsburgh, PA). Phalloidin-iFluor 488 was purchased from Abcam (Cambridge, United Kingdom). The surfactant Hypermer B246 (98%) was received as a sample from Croda (Goole, United Kingdom). Eosin solution was purchased from Acros Organics.

### 2.2 Manufacturing and characterization of porous polyHIPE scaffolds

#### 2.2.1 Synthesis and methacrylation of 4PCL-M

4-arm polycaprolactone (4PCL) was synthesised via a previously detailed ring-opening polymerisation reaction (Aldemir Dikici et al., 2019). Briefly, ε-caprolactone (0.705 mol) and pentaerythritol (0.088 mol) were added to a three-neck flask under nitrogen flow, and the system was heated to 160 °C using an oil bath while being mixed at 200 rpm. When the pentaerythritol was completely dissolved, a drop of tin (II) 2-ethyl hexanoate was added as a catalyst, and the system was left covered with foil for 24 h. The system, now containing 4PCL, was then removed from the oil bath and left to cool down.

For methacrylation, the 4PCL was dissolved in sufficient DCM and then transferred to a 3-neck round flask. TEA (0.705 mol) was added along with an extra volume of DCM to ensure all reagents had dissolved. The system was immersed in an ice bath and stirred at 350 rpm. MEA (0.705 mol) was mixed with DCM, and the mixture was transferred to a dropping funnel (∼1 drop/s) and added to the ice bath system. After the MEA had been completely dispensed, the system was left overnight at room temperature in dark conditions and stirred at 350 rpm. The resulting mixture was then washed three times with 30 mM hydrochloric acid solution to remove excess TEA, MEA, and any salts that had formed. Most solvents were evaporated using a rotary evaporator and the resulting 4PCL-M was stored at −20 °C for later use.

#### 2.2.2 Synthesis and methacrylation of 80%-PGS-M

Equimolar weights of sebacic acid and glycerol were placed in a three-neck flask under nitrogen flow. The system was warmed to 120 °C in an oil bath and left for 24 h while being stirred at 150 rpm. The nitrogen source was then replaced with a vacuum and the system was left for a further 24 h. The system, now containing PGS prepolymer, was then removed from the oil bath and left to cool at room temperature.

For methacrylation, the prepolymer was dissolved completely in DCM and the system was immersed in an ice bath. TEA was added accordingly to achieve 80% PGS-M (1 g of PGS prepolymer possesses approximately 0.00387 mol of OH to be methacrylated19). MeHQ was added at a ratio of 1 mg per 1 g of PGS prepolymer to prevent undesired crosslinking. After mixing, MEA was added via a dropping funnel (∼1 drop/s). The system was covered with foil and left for 24 h while stirring at 150 rpm. Following this, 0.5 mg of MeHQ per 1 g of PGS prepolymer was added to stop crosslinking. The resulting mixture was washed 3 times with 30 mM HCl solution in a separating funnel to remove byproducts. Finally, solvents were evaporated using a rotary evaporator until the resulting 80%-PGS-M had a honey-like viscosity. The PGS-M was transferred to a bottle and stored at −20 °C for later use.

#### 2.2.3 Mechanical characterisation of bulk PCL-M and PGS-M blends

From this point onwards, 4PCL-M and 80%-PGS-M will be entitled ‘PCL-M’ and ‘PGS-M’ respectively, unless stated otherwise. Bulk monoliths of PCL-M and PGS-M blended in different ratios underwent tensile testing to determine the mixability of the two polymers. To make the bulk monoliths, 3.0 g of polymer blend was mixed with 4% (w/w) photoinitiator in a centrifuge tube. The mixture was centrifuged at 2,500 rpm for 5 min to remove bubbles. The mixture was then injected into a dog-bone-shaped PDMS mould based on a modified version of ASTM D638-10, and crosslinked via exposure to UV light for 5 min until solidified. Dog-bone samples had a thickness of 2.70–3.00 mm and a width of 1.55–1.77 mm. Tensile testing of the scaffolds was completed using a MultiTest-dv tester (Mecmesin, Slinfold, United Kingdom) equipped with a 250 N load cell, a grasp distance of 10 mm, and an extension rate of 1 mm/min.

#### 2.2.4 Surface wettability

Water contact measurements were used to analyse the hydrophilicity of the 50:50 PCL-M:PGS-M blend in comparison to PCL-M and PGS-M individually. Bulk scaffolds were prepared in the same way as described in Section 2.2.3. The sessile drop method with deionised water was used to measure the contact angle using a contact angle goniometer (Goniometer FTA 200). The mean contact angle was calculated from three different samples, with two different surface locations on each.

#### 2.2.5 Preparation of PCL-M:PGS-M blended polyHIPEs

The desired weights of PCL-M and PGS-M were added into a glass vial, according to the target ratio, to obtain a total polymer weight of 0.5 g. Then, 0.05 g of Hypermer surfactant (10% (w/w)) was added. The mixture was heated with a heat gun until the surfactant was completely melted. The mixture was then allowed to reach room temperature, and 0.5 g of a 40:60 (w/w) chloroform/toluene solvent blend was added. The mixture was stirred using a magnetic stirrer at 500 rpm for 5 min in a water bath set at 37 °C. Once homogeneous, 2.5 mL of deionised water was added dropwise (∼1 drop/s) whilst the mixture continued to be subjected to stirring to form the emulsion. Whilst stirring continued, 5% (w/w) photoinitiator was added to the glass vial. Samples of the emulsion were transferred to glass microscopy slides and imaged using optical microscopy to visualise the liquid template. The emulsion was then transferred to a PDMS mould and placed under UV light using an OmniCure Series 1,000 (100 W, Lumen Dynamics, Canada) for 5 min on each side.

#### 2.2.6 Tailoring of PCL-M:PGS-M blended polyHIPE porous structure

PolyHIPEs were prepared using a 50:50 blend of PCL-M and PGS-M to attain desirable mechanical properties. The method used was the same as described in Section 2.2.5, but the conditions were varied according to Table 1 to assess the impact on the resulting porous structure.

**TABLE 1:** The varying parameters used to make the PCL-M:PGS-M blend polyHIPEs that were assessed to create scaffolds with ideal porous structures for tissue-engineered applications.

Sample reference	Water bath temperature (°C)	Chloroform %	Toluene %	Added water content (mL)
*Base*	37	40	60	2.5
*37_4C:6T_4.0*	37	40	60	4.0
*37_4C:6T_4.5*	37	40	60	4.5
*50_4C:6T_4.0*	50	40	60	4.0
*50_6C:4T_4.0*	50	60	40	4.0
*50_2C:8T_4.0*	50	20	80	4.0
*50_8C:2T_4.0*	50	80	20	4.0

Using each set of conditions, 6 attempts were undertaken to form emulsions to assess the repeatability.

#### 2.2.7 Morphological investigation of polyHIPE scaffolds

SEM was used to analyse the changes in the porous structure of 50:50 PCL-M:PGS-M blends resulting from the tailoring process. Flat disc polyHIPE samples were briefly washed in acetone to remove uncrosslinked polymer, then washed in methanol at room temperature for 24 h. Samples were air dried at room temperature in a fume cupboard to allow residual solvents to evaporate. Samples were cut to reveal the internal structure and were gold sputter-coated in 15 kV for 5 min to increase conductivity. A FEI Inspect F SEM was used with 3 kV power. Three images were taken from each sample in different locations. Randomly, 50 pores from each image were selected and measurements were taken. Pores were selected by overlaying a 12 by 12 grid onto the image and measuring the diameter of pores which were in contact with the grid. A statistical correction factor of 2/√3 was applied to account for the underestimation of diameter caused by uneven sectioning (Dhavalikar et al., 2021; Barbetta and Cameron, 2004). The degree of interconnectivity was calculated by dividing the average window size, taken by measuring the windows of 50 randomly selected pores (selected as previously described), by the average pore size (d/D). Theoretical porosity was calculated using Equation 1, where v_i_ is the volume of the internal phase (water) and v_e_ is the volume of the external phase (PCL-M, PGS-M, surfactant, photoinitiator).

$$Porosity\;\left(\%\right)=\frac{v_{i}}{v_{i}+v_{e}}$$ (1)

#### 2.2.8 Mechanical characterization of blended HIPEs with different porous structures

To assess the tensile strength of the polyHIPE membranes, dog-bone-shaped samples were prepared using PDMS moulds based on a modified version of ASTM D638-10. The emulsions were injected into the moulds and then crosslinked via exposure to UV light for approximately 10 min (5 min on each side). A MultiTest-dv tester (Mecmesin, Slinfold, United Kingdom) was used with a 25 N load cell, a grasp distance of 10 mm, and an extension rate of 1 mm/min. Testing was completed at room temperature. Data on both force and extension were collected, and the Young’s modulus of each sample was calculated using the linear-elastic region of the generated stress-strain curve. Samples ranged in thickness from 2.21 to 2.46 mm.

### 2.3 Biological assessment of polyHIPE scaffolds

#### 2.3.1 Preparation of scaffolds for cell culture

Samples were prepared for cell culture using methods that have previously been reported (Johnson L et al., 2024; Munive-Olarte et al., 2025). Immediately after crosslinking, polyHIPE scaffolds were washed briefly in acetone to remove any uncured polymer, before being washed in methanol three times over 24 h to remove any remaining contaminants of surfactant and solvent. Scaffolds were then left in 70% ethanol for 24 h for sterilisation and transferred into PBS in sterile conditions. Three PBS washes were applied in 24 h. Before cell seeding, scaffolds were submerged in 1 mL of EM to replace the PBS from inside the pores. Finally, scaffolds were allowed to dry slightly in sterile conditions to encourage the absorption of cell suspension when applied.

#### 2.3.2 General cell culture and cell seeding

Two cell lines were used to assess the biological properties of PCL-M-only, PGS-M-only, and PCL-M:PGS-M polyHIPE scaffolds:

o MLOA5 cells - A late-stage murine osteoblast cell line (Kerafast, US).
o BJ5ta cells - A human immortalised fibroblast cell line (ATCC, US).

These two contrasting cell types were chosen to represent cell types typical of hard tissue (MLOA5 cells) and soft tissue (BJ5ta cells) to better assess the suitability of these scaffolds for multiple tissue engineering applications.

DMEM supplemented with 10% FBS, 2 mM L-glutamine, and 100 mg/mL penicillin/streptomycin was used as an expansion cell culture media (EM). The scaffolds were conditioned with EM for 1 h in the incubator in 24-well plates. Cells were defrosted from liquid nitrogen into separate T75 flasks and cultured until 90% confluency. The cells were expanded, trypsinized and counted using a haemocytometer. The cells were then centrifuged, and the cell pellets were resuspended in fresh EM (20,000 cells/20 μL). The media in the 24 well-plates was removed and 20 μL of the required cell suspension was homogeneously placed on the surface of each scaffold. Wells containing 1 mL of EM and no scaffold were used as control wells. The well plates were left in the incubator for 45 min to allow the cells to attach. After 45 min, 1 mL of EM was added to each well containing a scaffold and incubated overnight. This was considered to be day 0. EM was changed every 2–3 days.

#### 2.3.3 Cell attachment assay

A Quant-iT dsDNA high-sensitivity assay kit was used to find the seeding efficiency of MLOA5 cells and BJ5ta cells on PCL-M-only, PGS-M-only, and PCL-M:PGS-M scaffolds. 24 h after seeding, scaffolds were washed twice with PBS, and 1 mL of cell digestion buffer (10 mM Tris-HCL, 1 mM ZnCl2 and 1% Triton X-100 in distilled water) was added and incubated for 1 h at room temperature. Samples were then vortexed for 60 s and kept in the fridge at 4 °C overnight. The next day, three freeze-thaw cycles were applied (20 min at −80 °C, 15 min at 37 °C, 15 s vortex) to create the final lysate solutions. 10 μL of lysate was added to 90 μL of assay working solution in triplicate in black 96-well plates. The well plate was shaken for 10 s to ensure thorough mixing and then incubated for 10 min at room temperature to allow the DNA to fully conjugate. A plate reader was used to read fluorescence with an excitation wavelength of 485 nm and an emission wavelength of 535 nm. Cell-free scaffolds were used to account for any background fluorescence caused by the scaffolds themselves.

#### 2.3.4 Cell viability assay

Resazurin reduction (RR) assay was used to measure the cellular metabolic activity and therefore estimate the cell viability on the scaffolds at different time points. The assay is colourimetric whereby the nonfluorescent resazurin solution (blue) is reduced by the cells to form the fluorescent resorufin (pink). The presence of resorufin can be detected by a fluorescence plate reader. The resazurin stock solution was diluted to 100 μL in EM to make the resazurin working solution. The EM was removed from the wells and the scaffolds were transferred to a fresh well plate using sterile tweezers. This was to prevent cells that had fallen off the scaffold from contributing to the assay. 1 mL of resazurin working solution was added to the scaffolds and a control well and the well plate was wrapped in aluminium foil and left in the incubator for 4 h. From each scaffold, triplicate samples of 150 μL of the reduced solution were transferred to a 96-well plate. The fluorescence was measured in the plate reader at an excitation wavelength of 540 nm and an emission wavelength of 630 nm. The RR assay was performed on days 1, 4 and 8. Different cell-seeded scaffolds were set-up for each time point to negate the effects of resazurin exposure.

#### 2.3.5 Measuring collagen deposition

Sirius red (SR) staining was used to observe the deposition and attachment of collagen onto PCL-M:PGS-M scaffolds by BJ5ta cells and MLOA5 cells. SR is a strong anionic dye that binds to collagen molecules. Successful attachment of collagen to the scaffolds would be an indication of the scaffold’s ability to support extracellular matrix deposition. Cells were cultured in a differentiation medium (for MLOA5 cells: 50 μg/mL ascorbic acid 2-phosphate (AA2P) and 5 mM beta-glycerolphosphate, for BJ5ta cells: 50 μg/mL AA2P) to encourage the deposition of collagen and were cultured for up to 28 days. Culture medium was removed and scaffolds were washed twice with PBS. Scaffolds were then fixed with 1 mL of 3.7% formaldehyde (FA) for 30 min at room temperature and washed twice with PBS. SR powder was dissolved in saturated picric acid (1% (w/v)) and filtered to remove any particles. Scaffolds were submerged in 1 mL of SR solution and left for 18 h. The solution was removed, and scaffolds were washed with deionised water every 5 min with orbital shaking until the water remained clear. Scaffolds were submerged with a known volume of 0.2 M NaOH/MeOH (1:1) to destain the SR and left for 30 min at room temperature with orbital shaking. 150 μL of the destain solution in triplicates was transferred to a clear 96-well plate and the absorbance was read at 405 nm. A fresh scaffold was used at each time point. Acellular scaffolds were used as a negative control.

#### 2.3.6 Fluorescence imaging

For fluorescence staining, scaffolds were stained with Sudan Black before cell seeding to quench them. This is because previous experiments showed that several fluorescent stains attached to the scaffolds themselves, making cell imaging difficult. On day 4, samples were fixed with 1 mL of 3.7% FA for 30 min and washed twice with PBS gently so as not to disturb the cells. Samples were then submerged in 0.1% (v/v) Triton X-100 (in PBS) for 20 min to increase the permeability of the cells, and washed three times with PBS. To visualise the F-actin filaments in cells, phalloidin (FITC) solution was added (1:1,000 diluted in PBS from stock solution) to samples and incubated for 30 min in the dark. Samples were washed three times in PBS. The nuclei of the cells were stained with 4′,6-diamidino-2-phenylindole (DAPI) solution (diluted 1:1,000 in PBS) and incubated in the dark for 15 min. Samples were washed three times in PBS and imaged with an LSM880 AiryScan Confocal Microscope (ZEISS, Germany). FITC (green channel) was imaged with 488 nm laser excitation and a spectral detection of 495–634 nm. DAPI (blue channel) was imaged with 405 nm laser excitation and a spectral detection of 410–495 nm. Z-stack images (2,580–2,580 pixels) were obtained and projected to a single image to account for uneven surfaces.

#### 2.3.7 Histological analysis

Histological analysis was performed on PCL-M:PGS-M, PCL-M-only, and PGS-M-only polyHIPEs seeded with MLOA5 cells and BJ5ta cells for 7 days. This was used to assess infiltration of cells into the scaffolds. Samples were washed twice with PBS and then fixed with 3.7% w/v formaldehyde for 24 h. The samples were again washed with PBS then placed into tissue cassettes and processed for 18 h (Leica TP 1020 tissue processor, Leica Biosystems, United Kingdom). The samples were cut in half, perpendicular to the seeding surface, to reveal internal structure and embedded in wax and sectioned onto glass slides. The slides were stained using haematoxylin for 60 s and eosin for 2.5 min, washed with tap water, dehydrated via a series of washes in industrial methylated spirits (IMS) before finally being washed twice with xylene. The slides were then imaged using an optical microscope.

### 2.4 Statistical analysis

Statistical analysis was completed using statistical analysis software (GraphPad Prism, Version 8.4.3, CA, United States). All data was analysed using a one-way or two-way analysis of variance (ANOVA) followed by a Tukey multiple comparison test. Error bars on graphs indicate standard deviation and the number of technical repeats (n) and experimental repeats (N) are given in figure captions where relevant.

## 3 Results and discussion

### 3.1 PCL-M:PGS-M in different ratios

A 1:1 M ratio of sebacic acid to glycerol was selected for the synthesis of PGS prepolymer because this stoichiometry reduces the extent of chain branching and delays the onset of network formation during step-growth polymerisation (Zhang and Grinstaff, 2014). Increased chain branching has been reported to reduce the solubility of the prepolymer, and limit subsequent functionalisation, which were essential considerations in this study (Nagata et al., 1997). In addition, the equimolar formulation was essential for there to be a sufficient density of available hydroxyl groups to enable methacrylation.

The total reaction time to form PGS prepolymer has been shown to influence the number average molecular weight (M_n_) and dispersity index of the resulting polymer (Pashneh-Tala et al., 2018). For this study, a relatively short total reaction time of 48 h was selected to target a molecular weight that was comparable to that of PCL-M, and to limit polydispersity. Reducing the breadth of the molecular weight distribution decreases the contribution of high-molecular-weight species that disproportionately increase viscosity, thereby improving the processability and enabling emulsion templating to be used.

Within this study, methacrylation of both polymer components is required to enable UV-initiated photocrosslinking, so that each polymer is covalently incorporated into the final network. An alternative approach of methacrylating only one component and physically entrapping the second within the crosslinked matrix could be explored in the future. This was not undertaken within this study due to the potential for phase separation and leaching of the non-crosslinked component, which was of particular concern due to the highly porous nature of the final structures. We note, however, that since PGS is also thermally curable (Wang et al., 2002), a hybrid strategy combining a photocurable component with a thermally crosslinkable component may represent an interesting route for further investigation.

**FIGURE 1:**
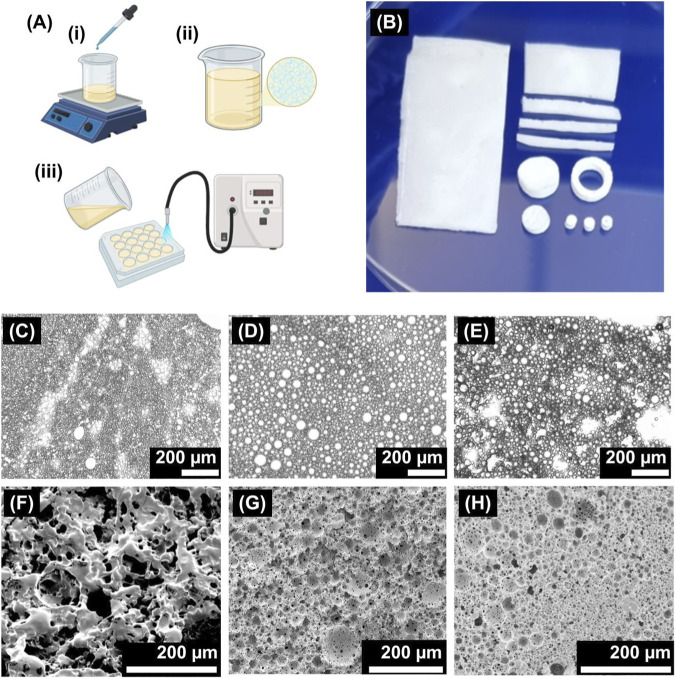
**(A)** The process of making polyHIPEs, (i) water is added dropwise to a polymer mixture with continued stirring to form (ii) a water-in-oil emulsion. (iii) The emulsion is then poured into a mould and crosslinked under UV light. **(B)** 50:50 PCL-M:PGS-M polyHIPEs are highly tailorable and can be formed into a wide range of shapes via emulsion templating or post-processing techniques. Optical microscopy images of the liquid template of PCL-M:PGS-M blended polyHIPEs in different ratios before blending: **(C)** 25PCL-M:75PGS-M, **(D)** 50PCL-M:50PGS-M, and **(E)** 75PCL-M:25PGS-M. Scanning electron micrographs of PCL-M:PGS-M blended polyHIPEs in different ratios: **(F)** 25PCL-M:75PGS-M, **(G)** 50PCL-M:50PGS-M, and **(H)** 75PCL-M:25PGS-M. Scale bars represent 200 μm.

PolyHIPEs were made using the conditions described in 2.2.5 (Figure 1). As discussed by Velazquez et al., the temporary stability of an emulsion, which is highly dependent on the solvents used, plays a decisive role in manufacturing HIPEs (Velázquez de la Paz et al., 2023). Chloroform and toluene were selected as a suitable polymer blend as chloroform aids the formation of larger pores, whilst toluene aids the dispersion of water droplets into the polymer phase (Velázquez de la Paz et al., 2023). The combined density of the polymers was matched with the ratio of solvents so that the density of the polymer phase supported the formation of round and interconnected droplets.

All polyHIPEs investigated in this study were photocrosslinked within 1 hour of emulsion preparation. This time window was selected to ensure that there was sufficient short-term emulsion stability prior to polymerisation. Optical microscopy of the liquid template (Figures 1C–E) reveals a clear difference in emulsion homogeneity as a function of blend composition. The 50:50 PCL-M:PGS-M blend produced a well-dispersed emulsion with rounded droplet morphology, which translates into a regular and interconnected porous architecture following curing. In contrast, formulations with asymmetric blend ratios exhibited reduced emulsion uniformity which is consistent with increased interfacial instability and phase segregation. These observations suggest that the balanced polymer composition promotes more favourable interfacial interactions during emulsification, thereby stabilising the liquid template. While stability limits were not examined in this study, a more comprehensive assessment of emulsion rheology and long-term stability would be valuable in future studies that are aimed at process optimisation and scale-up.

The PCL-M:PGS-M blend with 50:50 ratio of each polymer was selected as ideal due to its viscosity (medium to high) and its rounded pore morphology, with defined pores and interconnects as seen in Figure 1D.

### 3.2 Porous structure tailoring

**FIGURE 2:**
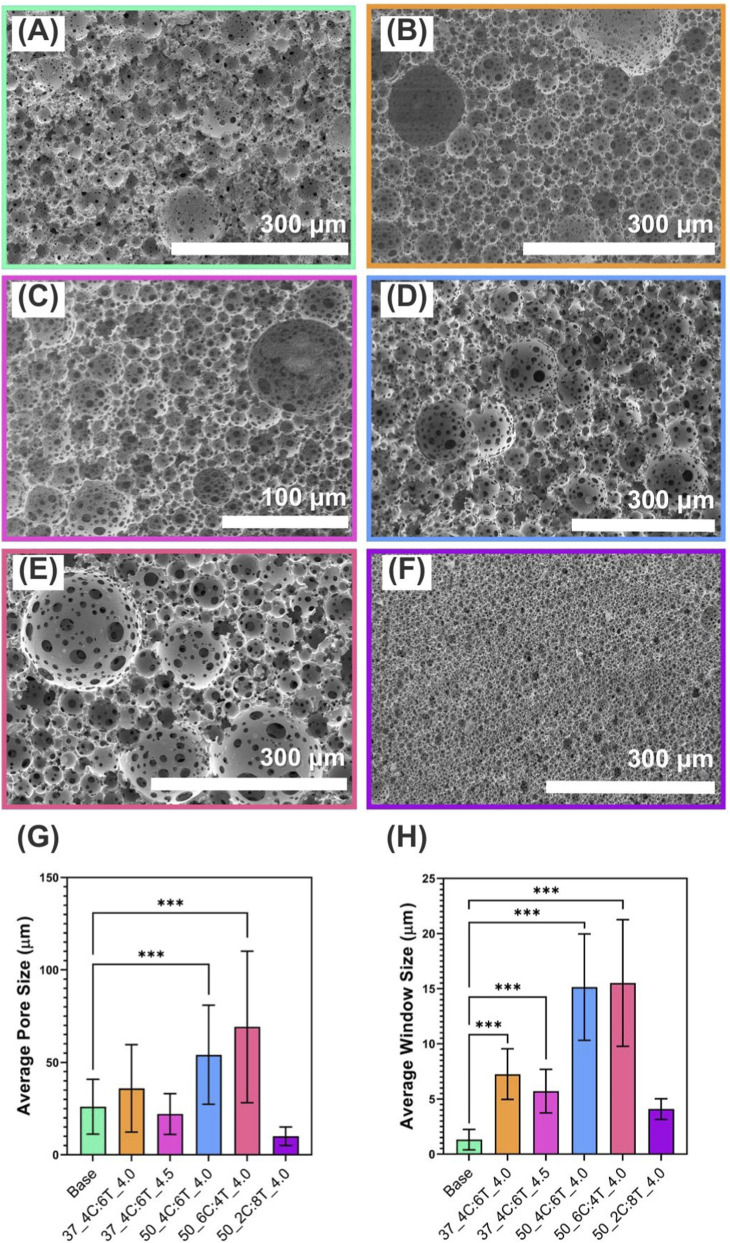
50:50 PCL-M:PGS-M porous scaffolds were fabricated using different parameters to investigate the effect on porous structure. Scanning electron micrographs of 50:50 PCL-M:PGS-M polyHIPEs: **(A)** Base, **(B)** 37_4C:6T_4.0, **(C)** 37_4C:6T_4.5, **(D)** 50_4C:6T_4.0, **(E)** 50_6C:4T_4.0, **(F)** 50_2C:8T_4.0. Changing the synthesis parameters affected **(G)** the mean pore size and **(H)** the mean window size (mean ± standard deviation, n = 50, ***P < 0.001).

In all cases, the external phase had a total weight of 1.05 g before addition of the internal phase. Increasing the water content resulted in an increased internal phase (with water volumes of 2.5 mL–4 mL) which resulted in higher theoretical porosities (Figure 2; Table 2). An increment in the mean pore size was also observed, from 26.0 ± 14.9 µm to 35.9 ± 23.6 μm at 2.5 and 4 mL of water respectively. This is likely because the higher the internal phase, the higher the frequency of contact between water droplets, increasing the risk of coalescence.

**TABLE 2:** Results of pore size analysis for 50:50 PCL-M:PGS-M polyHIPEs made using different synthesis parameters. Porous structures were imaged using scanning electron microscopy and measurements were recorded using imageJ software (n = 50).

Sample reference	Average pore size (µm)	Maximum pore size (µm)	Minimum pore size (µm)	Average window size (µm)	Maximum window size (µm)	Minimum window size (µm)	Degree of interconnectivity
Base	26.04	107.61	4.43	1.32	7.24	0.49	5.07
37_4C:6T_4.0	35.92	165.15	8.18	7.25	14.34	2.74	20.18
37_4C:6T_4.5	22.10	77.10	7.95	5.72	12.42	1.76	25.88
50_4C:6T_4.0	54.10	163.30	11.86	15.14	32.06	6.12	27.99
50_6C:4T_4.0	69.15	246.55	20.42	15.52	32.12	4.63	22.44
50_2C:8T_4.0	10.03	45.78	2.52	4.09	6.95	1.34	40.78

The increase in added water content from 2.5 mL to 4 mL resulted in an increase in theoretical volumetric porosity from 81.3% to 87.4%. The maximum water content that could be added was found to be 4.5 mL, resulting in a theoretical porosity of 88.7%. However, the formation of emulsions was inconsistent with 50% of attempts resulting in separated phases due to coalescence of the internal phase. Increasing the added water content to 5 mL resulted in unstable emulsions with separated phases in all attempts. Hence, a practical maximum added water content of 4 mL was decided for further tailoring as a stable emulsion was formed during mixing in all of the attempts. It has been reported that theoretical porosity and measured porosity, as measured by helium pycnometry, may vary by as much as 9%, with measured porosity always being lower than the theoretical porosity (Johnson L et al., 2024).

Increasing the temperature of the water bath during polyHIPE formation from 37 °C to 50 °C resulted in an increase in mean pore size from 35.9 ± 23.6 µm to 54.1 ± 26.8 µm. Mean window size also increased from 7.3 ± 2.3 µm to 15.1 ± 4.8 µm. Other studies have reported larger pore sizes when the temperature of the aqueous phase specifically is increased (Akay et al., 2004; Carnachan et al., 2006). This is thought to be caused by an increase in the thermal agitation of the water droplets resulting in a higher frequency of contact between them and hence, a higher probability of droplet coalescence (Weiner, 1975). Additionally, heating of the emulsion as a whole, rather than of the internal phase only, causes the surfactant in the interfacial film separating water droplets to become more soluble in the continuous phase and migrate from the interface (Weiner, 1975). This, in turn, raises the interfacial tension and further promotes droplet coalescence. Increasing the temperature of the internal phase is also associated with decreasing the stability of the emulsion (Aldemir Dikici and Claeyssens, 2020). Under the tested conditions, an increasing temperature did not result in unstable emulsions. This was potentially aided by the solvent blend but could also be the result of other non-tested factors including the polymer composition and the inclusion of surfactant. Temperatures higher than 50 °C were not tested due to the inclusion of volatile solvents in the organic phase.

The solvents used to facilitate emulsion formation are known to affect the phase interactions. Previously it has been shown that in PCL-M and PGS-M polyHIPEs, the use of chloroform produces less stable emulsions with larger pores due to its high viscosity whereas toluene produces more stable emulsions with smaller pores due to its low interfacial tension (Velázquez de la Paz et al., 2023). Hence, in this study, chloroform and toluene were used in combination and different ratios were assessed to determine the ideal. It was observed that an increasing ratio of chloroform to toluene resulted in larger pores as expected. Emulsions formed with a solvent ratio of 20:80 chloroform:toluene had the smallest pores whereas those formed with a ratio of 60:40 chloroform:toluene had the largest. A ratio of 80:20 chloroform:toluene did not successfully form an emulsion in 100% of attempts (due to phase separation/coalescence) and hence was excluded from further study.

A point of consideration is that polyHIPE scaffolds made with porogenic solvents have been shown to shrink when dehydrated (Murphy et al., 2017; Pierre et al., 2006). Shrinkage is reversible upon rehydration; however all SEM imaging was completed using dehydrated scaffolds hence it is probable that measured pore sizes are smaller than they would be in a hydrated scaffold. In a clinical setting, implantation materials may be stored dry and then rehydrated prior to implantation. Adequate pre-hydration time can help ensure that the functional pore architecture that is required for cell infiltration and mass transport is achieved *in vivo*. Such considerations are important for the translational use of polyHIPE constructs.

Also of note is the impact of porosity on the degradation rate. Highly interconnected scaffolds with a high percentage porosity are associated with faster degradation than scaffolds with lower, less connected porosity due to increased permeability (Abbasi et al., 2020). Additionally, larger pores are also associated with a faster degradation rate due to the increased surface area (Wu and Ding, 2005). Although degradation rates were not assessed in this study, it can be predicted that PCL-M:PGS-M porous scaffolds will degrade at a faster rate than non-porous scaffolds made of the same blend. What is still not certain however, is the speed and mechanism of degradation for PCL-M:PGS-M porous scaffolds, and how it will differ from pure PCL-M and pure PGS-M porous scaffolds. The degradation of both polymers is hydrolytic, and given their relative hydrophobicity and crosslinked nature, macroscopic objects of both PCL-M and PGS-M typically undergo surface erosion (Zaky et al., 2014). Degradation via surface erosion is thought to be preferred for tissue engineering applications as mechanical properties are maintained throughout the process, unlike with bulk degradation (Field et al., 2021). Further investigation is required to assess the rate of degradation of PCL-M:PGS-M scaffolds *in vivo,* as well as the effect that degradation byproducts may have on local pH.

The ability to easily create polyHIPEs with a range of pore sizes is beneficial to a variety of tissue engineering applications. For example, porous scaffolds for bone tissue engineering have an optimal pore size range of 100–135 µm to stimulate significant bone growth (Murphy and O’Brien, 2010), however larger pores within the structure (>160 µm) are also needed for the vascularisation of constructs (Artel et al., 2011). Conversely, structures with small (<10 µm) yet frequent windows can be used as barrier membranes in which cellular interfaces are maintained whilst allowing for biochemical crosstalk (Chung et al., 2018). The present study demonstrated the broad range of available porous structures that can be made via emulsion templating of PCL-M:PGS-M blends. The structure with the largest pores (condition 50_6C:4T_4.0) had an average pore size of 69.1 ± 41.0 µm with the largest pore being measured at 257 µm whereas the structure with the smallest pores (condition 50_2C:8T_4.0) had an average pore size of only 10.0 ± 5.0 µm. Pore size could be increased further through a surfactant-free emulsion templating approach in which gelatin is used as the internal phase rather than water, however it should be considered that this approach requires additional post-processing steps to remove the gelatin from within the pores.

### 3.3 Mechanical characterisation

Due to the inconsistent ability to create stable emulsions, condition 37_4C:6T_4.5 was excluded from further analysis.

**FIGURE 3:**
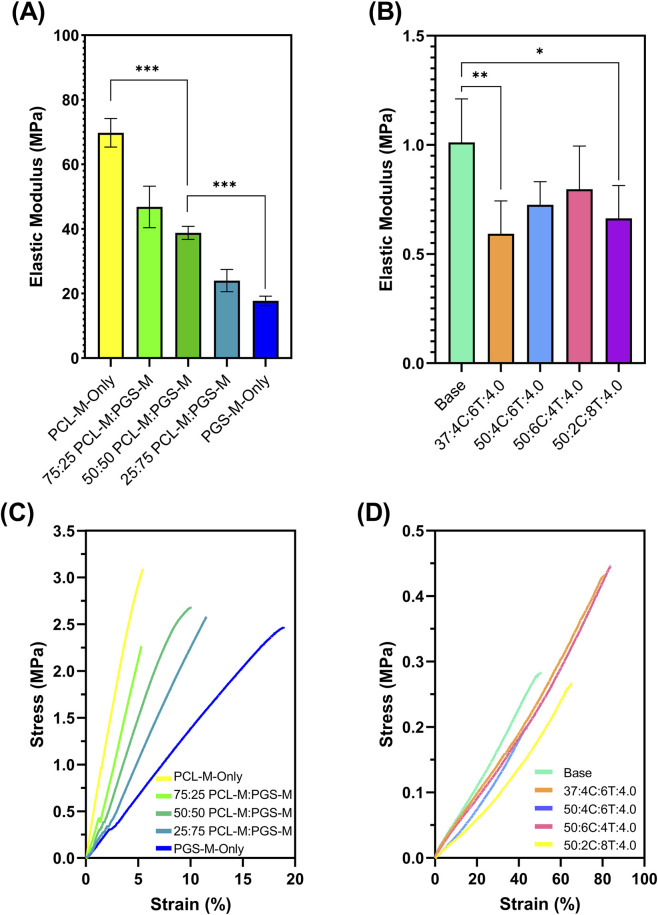
Tensile modulus of **(A)** bulk PCL-M and PGS-M in different ratios and **(B)** 50:50 PCL-M:PGS-M blend with different porous structures. Data are means ± standard deviation (n = 5; *P < 0.1, **P < 0.01, ***P < 0.001). Representative stress-strain curves for **(C)** bulk PCL-M and PGS-M in different ratios and **(D)** 50:50 PCL-M:PGS-M blend with different porous structures.

The miscibility of PCL-M and PGS-M was assessed through tensile testing of bulk samples created from different ratios of the two polymers. PCL-M-only and PGS-M-only bulk samples had tensile moduli of 69.8 MPa and 17.7 MPa respectively (Figure 3). The tensile moduli of both polymers can vary depending on the crosslinking density, hence the same polymer batch was used to create each sample and UV exposure time was kept constant (Pashneh-Tala et al., 2018). All ratios of the two polymers tested had tensile moduli which were in between those of the polymer-only values as expected. There was a linear relationship observed between the ratio of PCL-M to PGS-M and the tensile modulus demonstrating excellent miscibility of the two polymers under normal conditions. PCL-M and PGS-M blends were mixed manually with a spatula, although the subsequent centrifugation step to remove bubbles was also likely to have contributed to their mixing as both pre-polymers have similar molecular weights (2000–2,500 g/mol) (Dikici et al., 2020).

Whether this relationship continues in porous versions of the blends can be estimated by comparison to previously reported values. The tensile moduli of PCL-M-only and PGS-M-only polyHIPEs are reported as 2.4 ± 0.7 MPa (Jackson et al., 2023) and 0.24 ± 0.02 MPa (Furmidge et al., 2023) respectively. The unchanged 50:50 blend of the two materials gave a tensile modulus value of 1.0 ± 0.2 MPa, which lies close to the mean of the two polymers alone. This further confirms the miscibility of PCL-M and PGS-M demonstrating the potential to create porous scaffolds with highly tuneable mechanical properties by adjusting the ratio of the polymer blend.

Increasing the internal phase from 2.5 mL to 4.0 mL caused a lower tensile modulus from 1.01 ± 0.20 MPa to 0.59 ± 0.15 MPa due to the increase in theoretical porosity from 81.3% to 87.4%. Similarly, ultimate tensile strength (UTS) was also lower at higher internal phase volumes increased from 2.5 mL to 4 mL, with the average UTS recorded at 0.47 ± 0.41 MPa and 0.18 ± 0.09 MPa respectively. PolyHIPEs that had a larger average pore size but equal internal phase volumes had an increased tensile modulus and UTS. This is likely due to the increased wall thickness between pores (Aldemir Dikici et al., 2019; Lin-Gibson et al., 2007; Jiang et al., 2007).

### 3.4 Biological assessment

#### 3.4.1 Cell attachment

Of the assessed conditions, 50_6C:4T_4.0 produced the porous structure with the largest pores and windows, had an excellent degree of interconnectivity, and produced stable emulsions consistently. Hence this set of parameters was used to produce PCL-M:PGS-M blend polyHIPEs for biological assessment. It should be noted, however, that other porous structures may perform better for other tissue engineering applications (e.g., barrier layers). This is of particular note because pore geometry within tissue engineered scaffolds has been shown to impact cell behaviors such as attachment, proliferation, and differentiation (Mukasheva et al., 2024). This further highlights the advantage of using the blend, as it is possible to easily tailor the porous structure in various ways as required.

**FIGURE 4:**
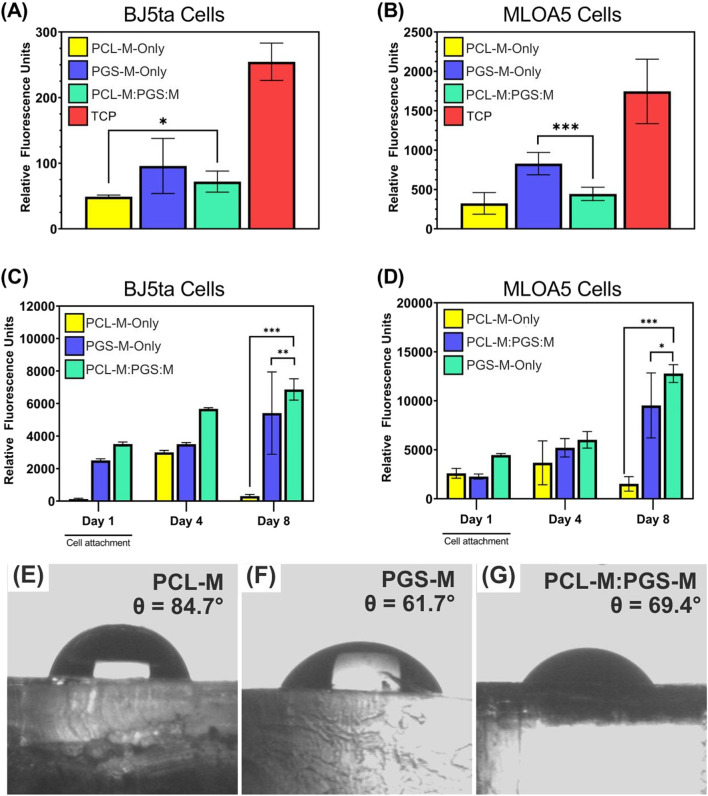
Results of DNA assay used to determine the attachment of **(A)** BJ5ta cells and **(B)** MLOA5 cells onto PCL-only, PGS-M-only, and PCL-M:PGS-M porous scaffolds after 24 h compared to tissue culture plate monolayer (N = 3, n = 3, *P < 0.05 and ***P < 0.001). Results of resazurin assay from **(C)** BJ5ta cells and **(D)** MLOA5 cells seeded onto PCL-M-only, PGS-M-only, and PCL-M:PGS-M porous scaffolds on day 1, 4, and 8 (N = 3, n = 3; *P < 0.05, **P < 0.01, and ***P < 0.001). Representative water contact angle images of **(E)** bulk PCL-M, **(F)** bulk PGS-M, and **(G)** bulk 50:50 PCL-M:PGS-M.

Cell attachment was assessed 24 h after cell seeding using a DNA quantification kit. For this experiment, the attachment of soft-tissue and hard-tissue cell types were assessed using BJ5ta cells and MLOA5 cells respectively, on PCL-M-only, PGS-M-only, and PCL-M:PGS-M porous scaffolds. As seen in Figures 4A,B, DNA was found on all scaffolds after 24 h. In all cases, however, the amount present was significantly lower compared to the tissue culture plastic control. This is likely because the porosity within the scaffolds causes some of the cell suspension to drain through onto the tissue culture plastic. For BJ5ta cells, PCL-M:PGS-M scaffolds had significantly more attachment than PCL-M-only scaffolds, and there was no significant difference between PCL-M:PGS-M and PGS-M-only. For MLOA5 cells, however, PGS-M-only exhibited significantly more cell attachment than PCL-M:PGS-M.

Figures 4C,D show that on day 1, both BJ5ta cells and MLOA5 cells had a slight preference for the PCL-M:PGS-M blend although there were no significant differences between results. During cell seeding, it was observed that the cell suspension was completely absorbed into both the PGS-M-only and PCL-M:PGS-M blend scaffolds; however the cell suspension sat atop the PCL-M-only scaffolds demonstrating its hydrophobicity. It is likely that cells were still able to attach to the PCL-M-only scaffolds due to them being primed with media before cell seeding.

The hydrophilicity of the blend compared to PCL-M only and PGS-M was confirmed via water contact angle assessment, shown in Figures 4E–G. The water contact angle for PCL-M, PGS-M, and the 50:50 PCL-M:PGS:M blend was 84.7°, 61.7°, and 69.4° respectively, demonstrating that the blend was more hydrophilic than PCL-M only and therefore more favourable for supporting cell attachment.

Differences between the results of the DNA quantification assay and metabolic activity assay can likely be explained by the fact that the DNA assay includes DNA from dead cells. The larger pores of the PCL-M:PGS-M scaffolds may have made it easier to flush dead cells from the scaffolds before starting the assay, hence resulting in a lower measured DNA content.

#### 3.4.2 Cell growth and viability

Resazurin reduction assay was used to measure the metabolic activity of BJ5ta cells and MLOA5 cells at day 4 and day 8 to assess growth and viability on PCL-M-only, PGS-M-only, and PCL-M:PGS-M porous scaffolds. Figures 4C,D shows that on day 4, both cell types on all scaffolds had a higher metabolic activity compared to their results for day 1, indicating growth over time. From day 4–8, cell metabolic activity on PGS-M-only and PCL-M:PGS-M scaffolds increased but decreased significantly on PCL-M-only scaffolds for both cell types. Although this could be an indication of cytotoxicity from the PCL-M-only scaffolds, it is much more likely to be a result of its hydrophobicity because PCL-M-only polyHIPE scaffolds have previously been demonstrated to be non-cytotoxic over a longer timeframe (Dikici et al., 2020). It is probable that cells reached confluence over the top surface of the scaffold and, being unable to infiltrate into the pores of the scaffold due to hydrophobic limitations, detached as a cell layer. PCL-M is known to be a hydrophobic polymer and it is often recommended that scaffolds made from it should be surface treated to improve polymer-cell interaction and the adherence of ECM deposition (Aldemir Dikici et al., 2019; Owen et al., 2016). Though the recorded water contact for our PCL-M was below 90° (Figure 4E), it was still higher than that of both PGS-M and the blend. Metabolic activity was significantly higher for both MLOA5 cells and BJ5ta cells on PCL-M:PGS-M scaffolds on day 8, compared to PCL-M-only and PGS-M-only. Here it is demonstrated that the inclusion of PGS-M with PCL-M improves the hydrophilicity of scaffolds, hence also improving cell growth over time and medium-term viability of the scaffolds.

No obvious cytotoxic effects were observed as a result of potential toxic components leaching from the polyHIPE scaffolds. The methods used to wash polyHIPE scaffolds prior to cell seeding have been reported on previously, but due to the tunability of the polyHIPEs, a comprehensive assessment of any solvent residuals would be required in the future to ensure washing stages are suitable for the specific chosen porous structure.

#### 3.4.3 Collagen deposition

**FIGURE 5:**
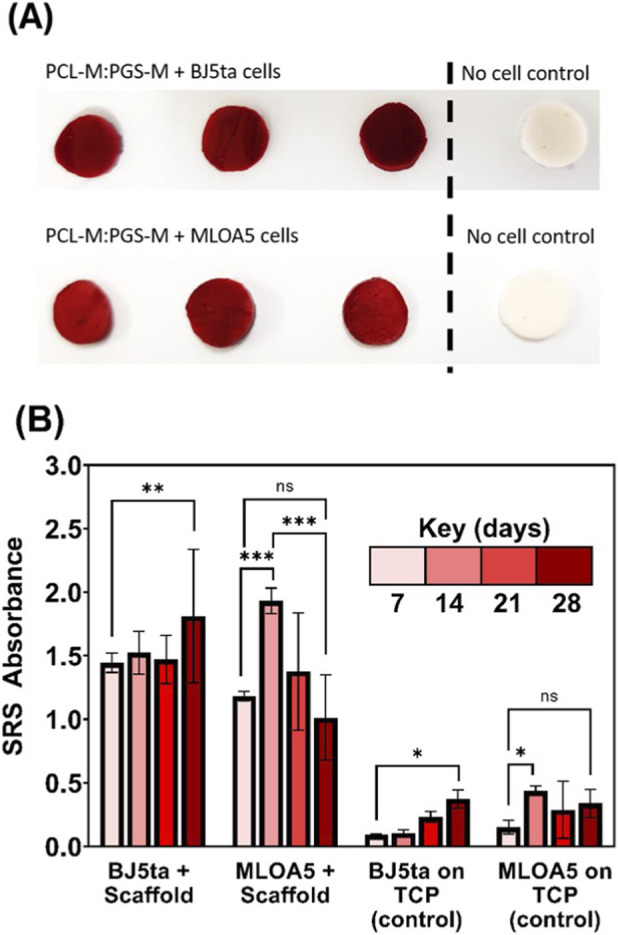
**(A)** Comparison of Sirius red staining (SRS) on PCL-M:PGS-M scaffolds seeded with BJ5ta cells and MLOA5 cells vs. acellular scaffolds after 14 days of culture. **(B)** SRS absorbance readings on scaffolds vs. on tissue culture plastic (TCP). (N = 3, n = 3; *P < 0.05, **P < 0.01). For all readings, the background colour (acellular controls) was subtracted.

Figure 5 shows that both MLOA5 cells and BJ5ta cells produced collagen onto the 50:50 PCL-M:PGS-M scaffolds readily and in higher quantities than in 2D culture. Notably collagen production by both cell types on the scaffolds was observed on day 7. Afterwards, there was not a significant increase in collagen quantity by BJ5ta cells until day 28. Conversely, collagen quantity deposited by MLOA5 cells significantly increased from day 7 to day 14, and then decreased thereafter. In 2D culture, it was visually observed that the majority of MLOA5 cells detached from the well plate on day 15 as a result of becoming over-confluent, and the cell sheet could be seen floating in the media. This is likely to have occurred in 3D culture too, causing some of the deposited collagen to be washed away during media changes, explaining the decrease on day 28 in Figure 5B, however further investigation is required to confirm this. BJ5ta cells remained attached in 2D culture for the full 28 days and produced large amounts of collagen on 3D scaffolds. The attachment of collagen to scaffolds could be further improved with treatments such as plasma coating.

The results of the SR assay indicate that the PCL-M:PGS-M scaffolds are able to support the deposition of ECM by these 2 cell types over the long term. ECM is the non-cellular component within tissues deposited by cells and is important for providing the structural support and biochemical cues required for adequate tissue regeneration. The interactions between cells and ECM are well known to play a critical role in regulating the repair of a variety of tissues (Kular et al., 2014; Wilgus, 2012; Diller and Tabor, 2022). The ability of PCL-M:PGS-M scaffolds to support strong attachment of deposited ECM makes them an appealing choice for ECM-based tissue engineering applications, such as decoration with decellularised ECM.

#### 3.4.4 Cell infiltration

**FIGURE 6:**
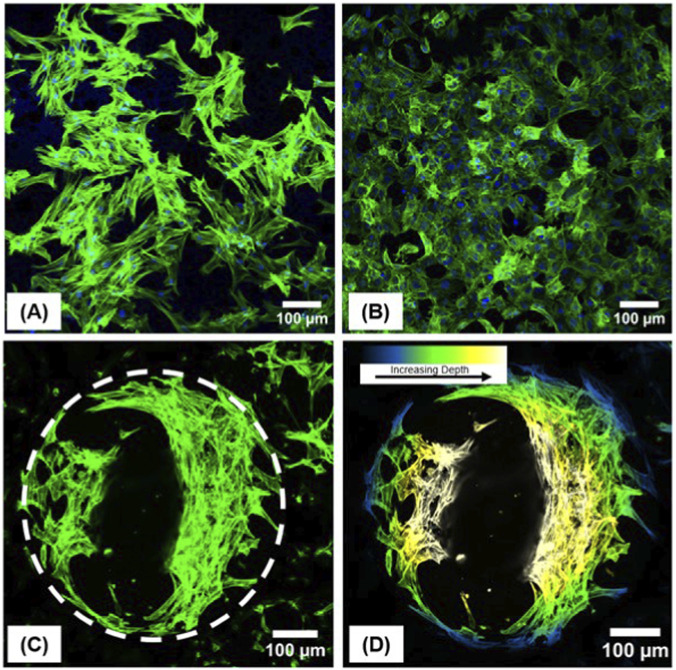
Fluorescent staining (blue: DAPI, green: Phalloidin FITC) of **(A)** BJ5ta cells and **(B)** MLOA5 cells on day 4 of culture on porous PCL-M:PGS-M scaffolds. BJ5ta cells were observed to grow into the largest pores of the scaffold as shown by **(C)** the pore outline and **(D)** the depth of the cells within it.

MLOA5 cells and BJ5ta cells were observed growing on PCL-M:PGS-M blended polyHIPE scaffolds using a Zeiss LSM 880 inverted microscope. The cell’s nuclei were stained with DAPI and the cell’s F-actin was stained with FITC-Phalloidin (Figure 6). Before cell seeding, scaffolds were stained with Sudan black, a non-cytotoxic dye. Previous experimental attempts demonstrated that DAPI actively stained the PCL-M:PGS-M scaffolds, making it difficult to visualise the cell’s nuclei against the also stained porous structure in the background. The PCL-M:PGS-M scaffolds were also readily stained by Sudan black, hence this was used to quench the scaffolds to prevent the DAPI stain from attaching to them during cell staining. Cell staining was performed on day 4, where cell numbers were lower to allow for clear visualisation of the cell morphology.

On day 4, MLOA5 cells were observed to have a cuboidal shape on PCL-M:PGS-M porous scaffolds which are typical of osteoblasts (Rutkovskiy et al., 2016) (Figure 6B). Meanwhile, BJ5ta cells were observed to have an elongated, spindle shape which is typical of fibroblasts (Ravikanth et al., 2011) (Figure 6A). Confocal microscopy revealed an even distribution of cells over the surface of the scaffolds demonstrating excellent cell attachment on PCL-M:PGS-M scaffolds without the need for surface modification, further supporting the cell assay results. As seen in Figure 6D, Z-stack images also revealed that cells grew into and lined the inner surfaces of pores within the scaffolds rather than spanning across them This confirms that the tailored porous structure is appropriate for tissue engineering applications requiring cellular invasion.

**FIGURE 7:**
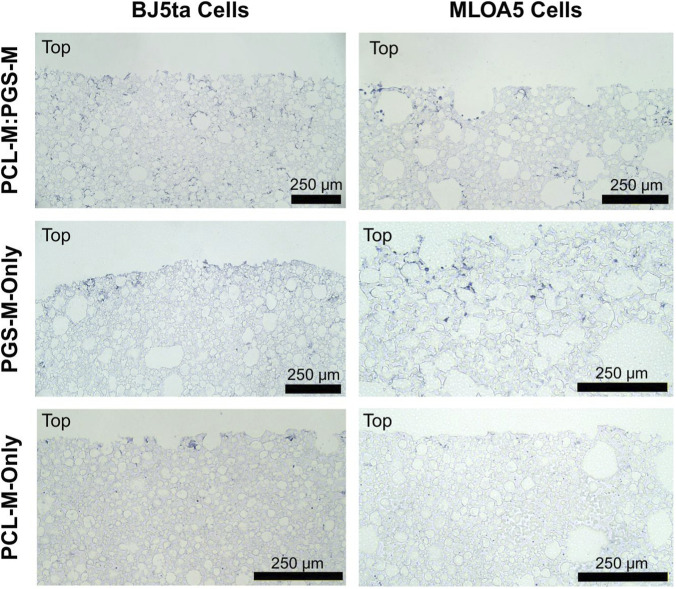
H&E staining of scaffold cross sections shows infiltration of BJ5ta and MLOA5 cells into PCL-M:PGS-M, PGS-M-only, and PCL-M-only polyHIPE scaffolds after 7 days of culture. Scale bars represent 250 μm. Magnification is ×10. Top of scaffolds (cell seeded surface) is indicated on images.

Infiltration of cells into polyHIPE scaffolds was further investigated via histological analysis. Figure 7 shows that both BJ5ta and MLOA5 cells were able to grow into the internal porous structure of the PCL-M:PGS-M scaffolds. Evidence of cells was demonstrated by the presence of purple deposits which could be seen across the depth of the scaffolds and were not observed on acellular scaffolds. It is likely that some cells attached to the bottom of the scaffolds during cell seeding and hence infiltration occurred from both faces of the scaffold, explaining why cells were able to span across in a short time frame. Histological analysis of PGS-M-only scaffolds showed infiltration from MLOA5 cells, but BJ5ta cells appear to have predominantly grown across the top surface with limited infiltration present. In comparison, PCL-M-only scaffolds had little-to-no areas of cellular presence at all from either cell type, further supporting the theory of cell detachment due to hydrophobicity.

Cellular infiltration is often an essential goal during biomaterial development as it demonstrates the ability of a material to provide the necessary environment to integrate with surrounding native tissue (Kim et al., 2020; Echeverria Molina et al., 2021). Well-interconnected scaffolds such as the PCL-M:PGS-M blend can facilitate accelerated tissue growth rates as well as permeability and cell-cell communication (Lutzweiler et al., 2020). The presence of cells across the depth of the blended scaffolds provides further evidence that the interconnected porous structure supports adequate mass transport, which is required to prevent areas of hypoxia resulting in necrotic cores (Maksoud et al., 2022). Overall, this further supports the decision to blend PCL-M and PGS-M to create scaffolds that are suitable for a variety of tissue engineering applications, as the individual polymers alone would require further tailoring or surface modification to achieve the same results.

## 4 Conclusion

In this study we have demonstrated that blending PCL-M and PGS-M provides a simple yet highly effective strategy to overcome the individual limitations of each polymer when used alone for tissue engineering applications. By combining the mechanical robustness of PCL-M with the inherent hydrophilicity and biological compatibility of PGS-M, we have developed tailorable polyHIPE scaffolds that exhibit stable, self-supporting porous structures following drying. Bulk blends of PGS-M and PCL-M in different ratios were mechanically tested and a direct relationship between an increasing percentage of PCL-M in the blend and an increase in tensile modulus was observed, demonstrating excellent miscibility and the ability to fine-tune mechanical properties for different tissue requirements. The porous structure of the 50:50 blend was shown to be tailorable through straightforward adjustments to the synthesis parameters, making it possible to create structures with high pore interconnectivity that can support cell infiltration and mass transport. Scaffolds supported cell attachment and medium-term viability without the need for post-fabrication surface modification. Overall, this work introduces a scalable and versatile blending-based methodology for fabricating highly interconnected polyHIPE scaffolds which offer a promising option for soft tissue engineering applications where cell ingrowth, nutrient exchange, and mechanical stability are important.

## Data Availability

The raw data supporting the conclusions of this article will be made available by the authors, without undue reservation.

## References

[B1] Abbasi N. Hamlet S. Love R. M. Nguyen N. T. (2020). Porous scaffolds for bone regeneration. J. Sci. Adv. Mater. Devices 5 (1), 1–9. 10.1016/j.jsamd.2020.01.007

[B2] Akay G. Birch M. A. Bokhari M. A. (2004). Microcellular polyHIPE polymer supports osteoblast growth and bone formation *in vitro* . Biomaterials 25 (18), 3991–4000. 10.1016/j.biomaterials.2003.10.086 15046889

[B3] Aldemir Dikici B. Claeyssens F. (2020). Basic principles of emulsion templating and its use as an emerging manufacturing method of tissue engineering scaffolds. Front. Bioeng. Biotechnol. 8, 875. 10.3389/fbioe.2020.00875 32903473 PMC7435020

[B4] Aldemir Dikici B. Sherborne C. Reilly G. C. Claeyssens F. (2019). Emulsion templated scaffolds manufactured from photocurable polycaprolactone. Polym. Guildf. 175, 243–254. 10.1016/j.polymer.2019.05.023

[B5] Aleemardani M. Trikić M. Z. Green N. H. Claeyssens F. (2022). Elastomeric, bioadhesive and pH-responsive amphiphilic copolymers based on direct crosslinking of poly(glycerol sebacate)-co-polyethylene glycol. Cite This Biomater. Sci. 10, 7015–7031. 10.1039/d2bm01335e 36342181 PMC9724602

[B6] Aleemardani M. Johnson L. Trikić M. Z. Green N. H. Claeyssens F. (2023). Synthesis and characterisation of photocurable poly(glycerol sebacate)-co-poly(ethylene glycol) methacrylates. Mater Today Adv. 19, 100410. 10.1016/j.mtadv.2023.100410

[B7] Artel A. Mehdizadeh H. Chiu Y. C. Brey E. M. Cinar A. (2011). An agent-based model for the investigation of neovascularization within porous scaffolds. Tissue Eng. Part A 17 (17–18), 2133–2141. 10.1089/ten.tea.2010.0571 21513462

[B8] Barbetta A. Cameron N. R. (2004). Morphology and surface area of emulsion-derived (PolyHIPE) solid foams prepared with oil-phase soluble porogenic solvents: span 80 as surfactant. Macromolecules 37 (9), 3188–3201. 10.1021/ma0359436

[B9] Barry J. J. A. Silva MMCG Shakesheff K. M. Howdle S. M. Alexander M. R. (2005). Using plasma deposits to promote cell population of the porous interior of three-dimensional Poly(D,L-Lactic acid) tissue-engineering scaffolds. Adv. Funct. Mater 15 (7), 1134–1140. 10.1002/adfm.200400562

[B10] Borzacchiello A. Gloria A. Mayol L. Dickinson S. Miot S. Martin I. (2011). Natural/synthetic porous scaffold designs and properties for fibro-cartilaginous tissue engineering. J. Bioact. Compat. Polym. 26 (5), 437–451. 10.1177/0883911511420149

[B11] Cameron N. R. (2005). High internal phase emulsion templating as a route to well-defined porous polymers. Polym. Guildf. 46 (5), 1439–1449. 10.1016/j.polymer.2004.11.097

[B12] Carnachan R. J. Bokhari M. Przyborski S. A. Cameron N. R. (2006). Tailoring the morphology of emulsion-templated porous polymers. Soft Matter 2 (7), 608–616. 10.1039/b603211g 32680240

[B13] Chung H. H. Mireles M. Kwarta B. J. Gaborski T. R. (2018). Use of porous membranes in tissue barrier and co-culture models. Lab. Chip 18 (12), 1671–1689. 10.1039/c7lc01248a 29845145 PMC5997570

[B14] Dhavalikar P. Shenoi J. Salhadar K. Chwatko M. Rodriguez-Rivera G. Cheshire J. (2021). Engineering toolbox for systematic design of PolyHIPE architecture. Polym. (Basel) 13 (9), 1479. 10.3390/polym13091479 34064400 PMC8124597

[B15] Dikici B. A. Dikici S. Reilly G. C. MacNeil S. Claeyssens F. (2019). A novel bilayer polycaprolactone membrane for guided bone regeneration: combining electrospinning and emulsion templating. Mater. (Basel) 12 (16). 10.3390/ma12162643 PMC6721100 31434207

[B16] Dikici A. Reilly G. C. Claeyssens F. (2020). Boosting the osteogenic and angiogenic performance of multiscale porous polycaprolactone scaffolds by *in vitro* generated extracellular matrix decoration. ACS Appl. Mater Interfaces 12, 12510–12524. 10.1021/acsami.9b23100 32100541 PMC7146758

[B17] Diller R. B. Tabor A. J. (2022). The role of the extracellular matrix (ECM) in wound healing: a review. Biomimetics 7 (3), 87. 10.3390/biomimetics7030087 35892357 PMC9326521

[B18] Echeverria Molina M. I. Malollari K. G. Komvopoulos K. (2021). Design challenges in polymeric scaffolds for tissue engineering. Front. Bioeng. Biotechnol. 9, 617141. 10.3389/fbioe.2021.617141 34195178 PMC8236583

[B19] Field J. Haycock J. W. Boissonade F. M. Claeyssens F. (2021). A tuneable, photocurable, Poly(Caprolactone)-Based resin for tissue engineering—synthesis, characterisation and use in stereolithography. Molecules 26 (5), 1199. 10.3390/molecules26051199 33668087 PMC7956195

[B20] Foudazi R. (2021). HIPEs to PolyHIPEs. React. Funct. Polym. 164, 104917. 10.1016/j.reactfunctpolym.2021.104917

[B21] Furmidge R. Jackson C. E. Velázquez de la Paz M. F. Workman V. L. Green N. H. Reilly G. C. (2023). Surfactant-free gelatin-stabilised biodegradable polymerised high internal phase emulsions with macroporous structures. Front. Chem. 11, 1236944. 10.3389/fchem.2023.1236944 37681209 PMC10481965

[B22] Ghasemi-Mobarakeh L. Prabhakaran M. P. Morshed M. Nasr-Esfahani M. H. Ramakrishna S. (2008). Electrospun poly(ɛ-caprolactone)/gelatin nanofibrous scaffolds for nerve tissue engineering. Biomaterials 29 (34), 4532–4539. 10.1016/j.biomaterials.2008.08.007 18757094

[B23] Gloria A. Russo T. D’Amora U. Santin M. De Santis R. Ambrosio L. (2020). Customised multiphasic nucleus/annulus scaffold for intervertebral disc repair/regeneration. Connect. Tissue Res. 61 (2), 152–162. 10.1080/03008207.2019.1650037 31398999

[B24] Goreninskii S. Yuriev Y. Runts A. Prosetskaya E. Plotnikov E. Bolbasov E. (2022). Pulsed vacuum arc deposition of nitrogen-doped diamond-like coatings for long-term hydrophilicity of electrospun poly (ε-caprolactone) scaffolds. Membranes 12 (11). 10.3390/membranes12111080 PMC9695898 36363635

[B25] He W. Ma Z. W. Yong T. Teo W. E. Ramakrishna S. (2005). Fabrication of collagen-coated biodegradable polymer nanofiber mesh and its potential for endothelial cells growth. Biomaterials 26 (36), 7606–7615. 10.1016/j.biomaterials.2005.05.049 16000219

[B26] Hollister S. J. (2005). Porous scaffold design for tissue engineering. Nat. Mater. 4 (7), 518–524. 10.1038/nmat1421 16003400

[B27] Huš S. Krajnc P. (2014). PolyHIPEs from methyl methacrylate: hierarchically structured microcellular polymers with exceptional mechanical properties. Polym. Guildf. 55 (17), 4420–4424. 10.1016/j.polymer.2014.07.007

[B28] Jackson C. E. Ramos-Rodriguez D. H. Farr N. T. H. English W. R. Green N. H. Claeyssens F. (2023). Development of PCL PolyHIPE substrates for 3D breast cancer cell culture. Bioeng. (Basel) 10 (5), 522. 10.3390/bioengineering10050522 37237592 PMC10215449

[B29] Jiang B. Wang Z. Zhao N. (2007). Effect of pore size and relative density on the mechanical properties of open cell aluminum foams. Scr Mater 56 (2), 169–172. 10.1016/j.scriptamat.2006.08.070

[B30] Johnson L D. V. Aleemardani M. Atkins S. Boissonade F. M. Claeyssens F. (2024). Emulsion templated composites: porous nerve guidance conduits for peripheral nerve regeneration. Mater Des. 239, 112779. 10.1016/j.matdes.2024.112779

[B31] Kim H. S. Kumbar S. G. Nukavarapu S. P. (2020). Biomaterial-directed cell behavior for tissue engineering. Curr. Opin. Biomed. Eng. 17, 100260. 10.1016/j.cobme.2020.100260 33521410 PMC7839921

[B32] Kramer S. Krajnc P. Pulko I. Kramer S. Krajnc P. Pulko I. (2023). Influence of monomer structure, initiation, and porosity on mechanical and morphological characteristics of thiol-ene PolyHIPEs. Macromol. Mater Eng. 308 (8), 2300010. 10.1002/mame.202300010

[B33] Kular J. K. Basu S. Sharma R. I. (2014). The extracellular matrix: structure, composition, age-related differences, tools for analysis and applications for tissue engineering. J. Tissue Eng. 5, 1–17. 10.1177/2041731414557112 25610589 PMC4883592

[B34] Li L. LaBarbera D. V. (2017). 3D high-content screening of organoids for drug discovery. Compr. Med. Chem. 8, 388–415. 10.1016/B978-0-12-409547-2.12329-7

[B35] Liang H. Y. Lee W. K. Hsu J. T. Shih J. Y. Ma T. L. Vo T. T. T. (2024). Polycaprolactone in bone tissue engineering: a comprehensive review of innovations in scaffold fabrication and surface modifications. J. Funct. Biomater. 15 (9), 243. 10.3390/jfb15090243 39330219 PMC11433047

[B36] Lin-Gibson S. Cooper J. A. Landis F. A. Cicerone M. T. (2007). Systematic investigation of porogen size and content on scaffold morphometric parameters and properties. Biomacromolecules 8 (5), 1511–1518. 10.1021/bm061139q 17381151

[B38] Lutzweiler G. Halili A. N. Vrana N. E. (2020). The overview of porous, bioactive scaffolds as instructive biomaterials for tissue regeneration and their clinical translation. Pharmaceutics 12 (7), 602. 10.3390/pharmaceutics12070602 32610440 PMC7407612

[B39] Mukasheva F. Adilova L. Dyussenbinov A. Yernaimanova B. Abilev M. Akilbekova D. (2024). Optimizing scaffold pore size for tissue engineering: insights across various tissue types. Front. Bioeng. Biotechnol. 12, 1444986. 10.3389/fbioe.2024.1444986 39600888 PMC11588461

[B40] Ma Y. Zhang C. Wang Y. Zhang L. Zhang J. Shi J. (2021). Direct three-dimensional printing of a highly customized freestanding hyperelastic bioscaffold for complex craniomaxillofacial reconstruction. Chem. Eng. J. 411, 128541. 10.1016/j.cej.2021.128541

[B41] Maksoud F. J. Velázquez de la Paz M. F. Hann A. J. Thanarak J. Reilly G. C. Claeyssens F. (2022). Porous biomaterials for tissue engineering: a review. J. Mater Chem. B 10 (40), 8111–8165. 10.1039/d1tb02628c 36205119

[B42] Mert H. H. Mert E. H. (2022). “Emulsion templated hierarchical macroporous polymers”, in Advanced functional porous materials. 43–86. Available online at: https://link.springer.com/chapter/10.1007/978-3-030-85397-6_3.

[B43] Montanheiro T. L. do A. Schatkoski V. M. de Menezes B. R. C. Pereira R. M. Ribas R. G. de Freitas A. d. S. M. (2022). Recent progress on polymer scaffolds production: methods, main results, advantages and disadvantages. Express Polym. Lett. 16 (2), 197–219. 10.3144/expresspolymlett.2022.16

[B44] Munive-Olarte A. Durgut E. Verbruggen S. W. Claeyssens F. Reilly G. C. (2025). Particle stabilised high internal phase emulsion scaffolds with interconnected porosity facilitate cell migration. Biomed. Mater. 20 (6), 065005. 10.1088/1748-605X/ae05de 40930136

[B45] Murphy C. M. O’Brien F. J. (2010). Understanding the effect of mean pore size on cell activity in collagen-glycosaminoglycan scaffolds. Cell Adh Migr. 4 (3), 377–381. 10.4161/cam.4.3.11747 20421733 PMC2958613

[B46] Murphy A. R. Ghobrial I. Jamshidi P. Laslett A. O’Brien C. M. Cameron N. R. (2017). Tailored emulsion-templated porous polymer scaffolds for iPSC-derived human neural precursor cell culture. Polym. Chem. 8 (43), 6617–6627. 10.1039/c7py01375b

[B47] Nagata M. Ibuki H. Sakai W. Tsutsumi N. (1997). Synthesis, characterization, and enzymatic degradation of novel regular network aliphatic polyesters based on pentaerythritol. Macromolecules 30 (21), 6525–6530. 10.1021/ma970686o

[B48] Ni R. Qian B. Liu C. Liu X. Qiu J. (2018). A cross-linking strategy with moderated pre-polymerization of resin for stereolithography. RSC Adv. 52, 29583–29588. 10.1039/c8ra05432k 35547328 PMC9085295

[B49] Owen R. Sherborne C. Paterson T. Green N. H. Reilly G. C. Claeyssens F. (2016). Emulsion templated scaffolds with tunable mechanical properties for bone tissue engineering. J. Mech. Behav. Biomed. Mater 54, 159–172. 10.1016/j.jmbbm.2015.09.019 26458114 PMC4717122

[B50] Pashneh-Tala S. Owen R. Bahmaee H. Rekštyte S. Malinauskas M. Claeyssens F. (2018). Synthesis, characterization and 3D micro-structuring via 2-photon polymerization of poly(glycerol sebacate)-methacrylate-an elastomeric degradable polymer. Front. Phys. 6 (MAY), 353015. 10.3389/fphy.2018.00041

[B51] Pashneh-Tala S. Field J. Fornesa B. Molins Colomer M. Jackson C. E. Balcells M. (2023). Versatile, elastomeric and degradable polyHIPEs of poly(glycerol sebacate)-methacrylate and their application in vascular graft tissue-engineering. Mater Today Adv. 20, 100432. 10.1016/j.mtadv.2023.100432

[B52] Patel A. Gaharwar A. K. Iviglia G. Zhang H. Mukundan S. Mihaila S. M. (2013). Highly elastomeric poly(glycerol sebacate)-co-poly(ethylene glycol) amphiphilic block copolymers. Biomaterials 34 (16), 3970–3983. 10.1016/j.biomaterials.2013.01.045 23453201 PMC4507745

[B53] Pierre S. J. Thies J. C. Dureault A. Cameron N. R. Van Hest J. C. M. Carette N. (2006). Covalent enzyme immobilization onto photopolymerized highly porous monoliths. Adv. Mater. 18 (14), 1822–1826. 10.1002/adma.200600293

[B54] Rai R. Tallawi M. Grigore A. Boccaccini A. R. (2012). Synthesis, properties and biomedical applications of poly(glycerol sebacate) (PGS): a review. Prog. Polym. Sci. 37 (8), 1051–1078. 10.1016/j.progpolymsci.2012.02.001

[B55] Ravikanth M. Soujanya P. Manjunath K. Saraswathi T. R. Ramachandran C. R. (2011). Heterogenecity of fibroblasts. J. Oral Maxillofac. Pathol. 15 (2), 247–250. 10.4103/0973-029X.84516 22529592 PMC3329689

[B56] Rutkovskiy A. Stensløkken K. O. Vaage I. J. (2016). Osteoblast differentiation at a glance. Med. Sci. Monit. Basic Res. 22, 95–106. 10.12659/msmbr.901142 27667570 PMC5040224

[B57] Sant S. Hwang C. M. Lee S. H. Khademhosseini A. (2011). Hybrid PGS-PCL microfibrous scaffolds with improved mechanical and biological properties. J. Tissue Eng. Regen. Med. 5 (4), 283–291. 10.1002/term.313 20669260 PMC2972380

[B58] Silverstein M. S. (2014). PolyHIPEs: recent advances in emulsion-templated porous polymers. Prog. Polym. Sci. 39 (1), 199–234. 10.1016/j.progpolymsci.2013.07.003

[B59] Tan Q. Li S. Ren J. Chen C. (2011). Fabrication of porous scaffolds with a controllable microstructure and mechanical properties by porogen fusion technique. OPEN ACCESS Int. J. Mol. Sci. 12, 12–904. 10.3390/ijms12020890 21541032 PMC3083679

[B60] Velázquez de la Paz M. F. Aleemardani M. Furmidge R. Pashneh-Tala S. Claeyssens F. (2023). Effect of solvent type on porous structure of emulsion templated poly(glycerol sebacate)-methacrylate. Mater Lett. 347, 134566. 10.1016/j.matlet.2023.134566

[B61] Viswanathan P. Johnson D. W. Hurley C. Cameron N. R. Battaglia G. (2014). 3D surface functionalization of emulsion-templated polymeric foams. Macromolecules 47 (20), 7091–7098. 10.1021/ma500968q

[B62] Vogt L. Ruther F. Salehi S. Boccaccini A. R. (2021). Poly(Glycerol sebacate) in biomedical applications—A review of the recent literature. Adv. Healthc. Mater 10 (9), e2002026. 10.1002/adhm.202002026 33733604 PMC11468981

[B63] Wang Y. Ameer G. A. Sheppard B. J. Langer R. (2002). A tough biodegradable elastomer. Nat. Biotechnol. 20 (6), 602–606. 10.1038/nbt0602-602 12042865

[B69] Weiner N. D. (1975). Emulsions and emulsion technology, part I. J. Pharm. Sci. 64 (8), 1434. 10.1002/jps.2600640846

[B64] Wilgus T. A. (2012). Growth factor–extracellular matrix interactions regulate wound repair. Adv. Wound Care (New Rochelle) 1 (6), 249–254. 10.1089/wound.2011.0344 24527314 PMC3623585

[B65] Wu L. Ding J. (2005). Effects of porosity and pore size on *in vitro* degradation of three-dimensional porous poly(D,L-lactide-co-glycolide) scaffolds for tissue engineering. J. Biomed. Mater Res. A 75 (4), 767–777. 10.1002/jbm.a.30487 16121386

[B66] Zaky S. H. Lee K. W. Gao J. Jensen A. Close J. Wang Y. (2014). Poly(Glycerol sebacate) elastomer: a novel material for mechanically loaded bone regeneration. Tissue Eng. Part A 20 (1–2), 45–53. 10.1089/ten.TEA.2013.0172 24020497

[B67] Zhang H. Grinstaff M. W. (2014). Recent advances in glycerol polymers: chemistry and biomedical applications. Macromol. Rapid Commun. 35 (22), 1906–1924. 10.1002/marc.201400389 25308354 PMC4415886

[B68] Zulkifli M. Z. A. Nordin D. Shaari N. Kamarudin S. K. (2023). Overview of electrospinning for tissue engineering applications. Polym. (Basel) 15 (11), 2418. 10.3390/polym15112418 37299217 PMC10255387

